# Clinical efficacy of closed reduction and percutaneous parallel K-wire interlocking fixation of first metacarpal base fracture

**DOI:** 10.1186/s13018-021-02600-5

**Published:** 2021-07-14

**Authors:** Wu Wang, Min Zeng, Junxiao Yang, Long Wang, Jie Xie, Yihe Hu

**Affiliations:** 1 Department of Orthopedics, Xiangya Hospital, Central South University (Hunan Engineering Research Center of Biomedical Metal and Ceramic Implants), No. 87 Xiangya Road, Changsha, 410008 Hunan China; 2 Department of Orthopedics, The Fifth Affiliated Hospital of Xinjiang Medical University, Urumqi, Xinjiang, 830011 China

**Keywords:** Closed reduction, K-wire fixation, First metacarpal base fracture, Bennett fracture, Rolando fracture

## Abstract

**Background:**

This study aimed to explore the clinical efficacy of treating a first metacarpal base fracture by closed reduction and percutaneous parallel K-wire interlocking fixation between the first and second metacarpals.

**Methods:**

Twenty patients treated by the abovementioned modified technique (modified technique group) and ten patients treated by the traditional percutaneous K-wire fixation technique (traditional technique group) from October 2015 to November 2019 at our institution were retrospectively reviewed. The patients’ average age was 38 years (range, 16–61 years). The mean follow-up period was 13 months (range, 10–18 months). At the final follow-up, the functional recovery of the injured hand was assessed and compared between the modified and traditional technique groups. In addition, the functional recovery of the injured hand was compared with that of the uninjured hand within the modified technique group.

**Results:**

All patients recovered well, with no cases of infection or nonunion. Compared with the traditional technique group, the modified technique group had a shorter operative time, lower postoperative visual analogue scale pain score, better effective range of motion score of the first carpometacarpal joint (Kapandji score), and had almost no need for auxiliary plaster fixation, enabling functional exercise to be started earlier. Within the modified technique group, the mean hand grip strength, pinch strength, and Kapandji score on the injured side did not significantly differ to the values on the uninjured side in both the extra-articular and intra-articular fracture subgroups. While the abduction and flexion–extension arcs of the thumb on the injured hand were significantly smaller than those on the uninjured hand in both the extra-articular and intra-articular fracture subgroups, the patients felt clinically well with respect to daily activities and strength.

**Conclusion:**

The percutaneous parallel K-wire and interlocking fixation technique is simple, effective, and economical for first metacarpal base fractures.

**Supplementary Information:**

The online version contains supplementary material available at 10.1186/s13018-021-02600-5.

## Background

First metacarpal base fractures account for 80% of thumb fractures and 20% of fractures involving the articular surface of the first carpometacarpal joint [1]. These fractures are generally classified into four types: extra-articular, Bennett, Rolando, and comminuted [2]. Rolando fractures are also a type of comminuted fracture; therefore, the latter two types are often grouped together.

Fractures at the base of the first metacarpal are often unstable and require surgical treatment [2]. Open reduction and internal fixation often damage the ligaments around the joint and affect the blood circulation and joint stability, leading to delayed fracture healing and postoperative cicatrization that affects joint function; therefore, closed reduction is considered superior to open reduction [3]. Wagner’s method is a classic technique of closed reduction and percutaneous K-wire fixation for Bennett fractures [4]; it can also be used for Rolando fractures [5]. Percutaneous cross K-wire fixation is often used for Rolando fractures and extra-articular fractures of the first metacarpal base [5, 6]. However, these procedures require placement of a K-wire across the first carpometacarpal joint to fix the first metacarpal with the trapezium, which may increase the risk of traumatic arthritis [7]. Additionally, the end of the K-wire is not fixed in these techniques, which cannot prevent rotation and loosening of the K-wire, which often leads to secondary displacement [8].

Johnson [9] reported an operative technique that fixes the first and second metacarpals with one or two K-wires for first metacarpal base fractures. Van Niekerk and Ouwens [10] subsequently applied a parallel K-wire fixation technique but claimed that the results were unsatisfactory. However, Greeven et al. [11] reported that percutaneous intermetacarpal parallel K-wire fixation was safe and effective in the treatment of both intra-articular and extra-articular fractures of the first metacarpal base.

Our operative technique is a modification of the abovementioned procedures. Herein, we introduce this modified procedure and evaluate its efficacy in the treatment of first metacarpal base fractures.

## Methods

### Patients and clinical materials

The present study was approved by the ethics committee of our hospital. All operations were performed at our institution from October 2015 to November 2019 by the same surgical team. Among the patients with closed fractures at the base of the first metacarpal, 20 patients underwent the modified percutaneous intermetacarpal K-wire fixation technique (modified technique group: 11 extra-articular fractures and nine intra-articular fractures) and ten patients underwent the traditional percutaneous K-wire fixation technique (traditional technique group: five extra-articular fractures and five intra-articular fractures). The study population comprised 29 male patients and one female patient, and their average age was 38 years (range, 16–61 years). The fractures were present in 24 right hands and six left hands, and all patients were right-hand dominant. Nineteen patients were injured after falling, and 11 were injured while fighting (Tables 1 and 2). Radiographs were performed in all patients before admission, and the fractures were classified using the classic method established by Green and O’Brien (type I: Bennett fracture, type II: Rolando fracture, type IIIA: transverse extra-articular fracture, type IIIB: oblique extra-articular fracture, and type IV: epiphyseal fracture) [10]. Sixteen patients had a first metacarpal base fracture that did not involve the joint surface (type IIIA, *n* = 6; type IIIB, *n* = 10), nine had a Bennett fracture (type I), and five had a Rolando fracture (type II) (Tables 1 and 2). Follow-up included regular radiographs and clinical visits at 4, 6, and 12 weeks postoperatively. Clinical follow-up continued for an average of 1 year after surgery, while radiographs were not routinely obtained at 1 year postoperatively due to the reluctance of the patients to be exposed to radiation.

**Table 1:** Patient characteristics in the modified technique group

No.	Sex	Age	Fracture side	Trauma mechanism	Fracture type	Operation time (min)	Cast	K-wire removal (days)	Complication
1	M	38	R	Fall	Bennett	22	No	40	None
2	M	34	L	Fighting	Bennett	15	No	55	None
**3**	M	28	R	Fall	Rolando	25	Yes	60	None
4	M	36	R	Fighting	Extra transverse	20	No	42	None
**5**	M	44	R	Fall	Extra oblique	10	No	50	None
6	M	61	R	Fall	Extra oblique	18	No	36	None
7	M	45	R	Fall	Rolando	30	Yes	56	None
**8**	M	30	L	Fighting	Bennett^a^	35	No	58	None
9	M	22	R	Fighting	Extra transverse	11	No	53	None
10	M	16	R	Fall	Bennett	16	No	35	None
11	M	26	R	Fall	Bennett	13	No	38	None
12	M	42	R	Fall	Extra oblique	14	No	40	None
13	M	33	L	Fighting	Extra oblique	20	No	45	None
14	M	37	R	Fall	Bennett	23	No	52	None
15	F	40	R	Fall	Extra transverse	15	No	54	None
16	M	50	L	Fall	Extra oblique	18	No	48	None
17	M	43	R	Fall	Extra oblique	12	No	44	None
18	M	35	R	Fighting	Rolando	22	No	50	None
19	M	38	R	Fall	Extra oblique	15	No	49	None
20	M	20	R	Fighting	Extra transverse	12	No	47	None

Average age: 35.9 ± 10.6 years; average operation time: 18.3 ± 6.5 min; average K-wire removal time 47.6 ± 7.4 days

^a^Multi-fracture patient; representative cases are Nos. 3, 5, and 8

**Table 2:** Patient characteristics in the traditional technique group

No.	Sex	Age	Fracture side	Trauma mechanism	Fracture type	Operation time (min)	Cast	K-wire removal (days)	Complication
21	M	36	R	Fighting	Extra transverse	25	No	42	None
**22**	M	45	R	Fall	Rolando	45	Yes	58	Anchylosis
**23**	M	34	L	Fighting	Bennett	40	Yes	55	None
24	M	60	R	Fall	Extra oblique	30	Yes	52	None
25	M	41	R	Fall	Bennett	35	Yes	48	None
26	M	28	R	Fighting	Bennett	38	Yes	46	None
27	M	50	R	Fall	Rolando	46	Yes	60	Anchylosis
28	M	42	L	Fall	Extra transverse	32	Yes	50	None
29	M	35	R	Fighting	Extra oblique	22	Yes	46	None
30	M	40	R	Fall	Extra oblique	30	No	45	None

Average age: 41.1 ± 9.1 years; average operation time: 34.3 ± 8.0 min; average K-wire removal time: 50.2 ± 5.9 days

Representative cases are Nos. 22 and 23

### Surgical techniques and postoperative management

**Fig. 1:**
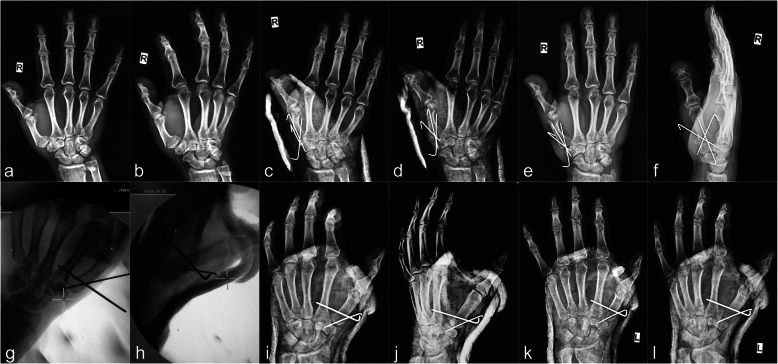
Representative cases of the traditional technique of percutaneous K-wire fixation. **a–f** Preoperative and postoperative follow-up radiographs of Patient 22. **g–l** Intraoperative fluoroscopy and postoperative follow-up radiographs of Patient 23

**Fig. 2:**
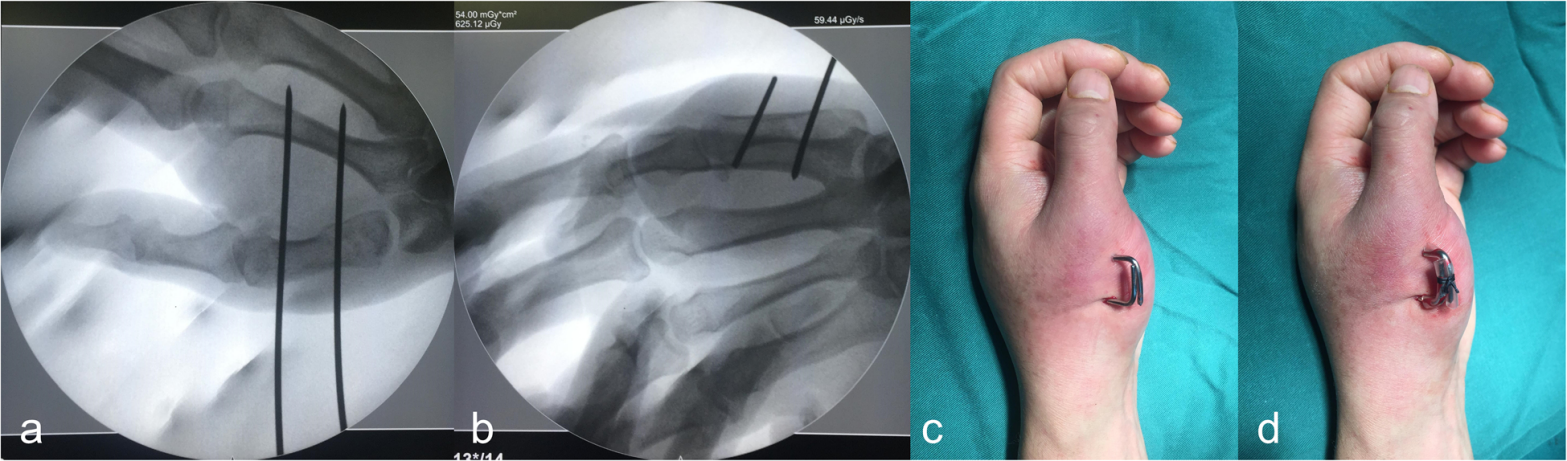
Intraoperative fluoroscopy and the operative area appearance of the patient treating by modified technique of percutaneous parallel K-wire interlocking fixation. **a, b** Intraoperative fluoroscopic images of Patient 5. **c, d** Operative area appearance of Patient 8

The procedure was performed under a brachial plexus block, and the injury-to-surgery interval ranged from 24 to 72 h. The patient was placed on the operating bed with the injured limb outspread on the radioparent side table and the forearm in pronation. Firstly, in both the traditional and modified surgical procedures, the assistant applied axial traction to restore the length of the first metacarpal. Abduction and pronation of the thumb were then applied to rectify the rotation displacement of the metacarpus. Most extra-articular fractures were satisfactorily reduced. For some fractures, however (especially Bennett and Rolando fractures), the surgeon needed to push the radial aspect of the first metacarpal base to assist in the reduction [8]. Secondly, in the traditional surgical procedure, multiple 1.0-mm K-wires were used to cross fix both ends of the fracture; in most cases, the wires inevitably passed through the first carpometacarpal joint (Fig. 1a–f). The other method used in the traditional technique group was Wagner’s method [4], in which a 1.0-mm K-wire was used to fix the distal end of the fracture and pass through the first carpometacarpal joint, and the first and second metacarpals were then fixed with another traverse K-wire at the distal end of the fracture (Fig. 1g–l). In the modified surgical procedure, a 1.6-mm K-wire was vertically drilled through the bases of the first and second metacarpals close to the distal end of the fracture, and another 1.6-mm K-wire was then drilled through the necks of the first and second metacarpals parallel to the first K-wire to strengthen the fixation (Fig. 2a, b). Thirdly, the ends of the K-wires were bent at 90° toward each other, and the wires were sheathed with a section of infusion tube. The wires were then strapped and fixed with silk thread to increase their stability and prevent rotation (Fig. 2c, d). This process is shown in more detail in Additional files 1, 2 online. Fracture reduction was evaluated by fluoroscopy [12]. The criteria for acceptable fracture reduction were a < 2-mm step of the articular surface and a < 2-mm space between fracture blocks [13].

**Fig. 3:**
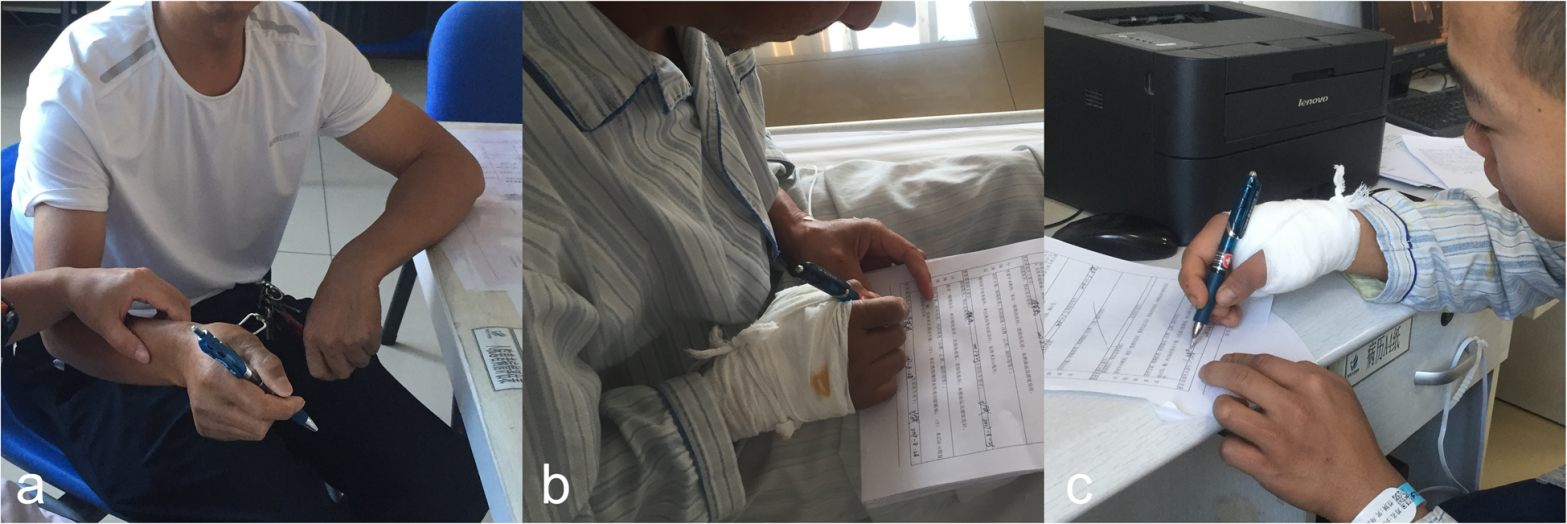
Functional exercises were performed immediately after modified surgery technique. The patients’ daily life, including eating and writing, was unaffected. **a, b** Patient 5. **c** Patient 9

No patients received antibiotics postoperatively because antibiotics were used intraoperatively. The K-wires outside the skin were sterilised on postoperative day 2 and then once every 1 to 2 weeks unless the dressing was contaminated. Postoperative stability was evaluated by the thumb activity; only two Rolando fractures in the modified technique group required postoperative plaster fixation, and only two extra-articular fractures in the traditional technique group were not fixed with plaster. Thumb flexion and extension exercises were performed immediately postoperatively, and the patients were able to perform simple pinching activities such as eating with tableware and writing with a pen in the modified technique group (Fig. 3); the patients with a plaster could only perform such activities when the plaster was removed.

### Evaluation

Clinical data were collected preoperatively, postoperatively, and during follow-up. Evaluation measures compared between the modified and traditional technique groups included the operative time, K-wire removal time, complications, visual analogue scale (VAS) score (0–10, where 0 = no pain), and effective range of motion score of the first carpometacarpal joint (Kapandji score) (0–10, where 10 = best) [14]. In the modified technique group, the subjective force of the injured hand (0–10, where 10 = best), grip and pinch strength of the injured thumb, and abduction and flexion–extension arc of the injured thumb were compared with the uninjured hand. The pinch and grip strength of the hand were measured by a dynamometer (Kayser Italia, Livorno, Italy) and were assumed to be 6% higher on the dominant than non-dominant side [15]. The range of thumb motion was measured by a goniometer.

All patients were followed up by the same senior orthopaedic surgeon, and radiographs were taken at 4 weeks to evaluate the fracture healing and determine the time of K-wire removal. Radiographs were then taken every 2 months until the fracture healed.

### Statistical analysis

Quantitative variables are presented as mean and standard deviation. The independent samples t test was used to compare the modified and traditional technique groups, and the paired samples t test was used to compare the functional test results between the injured and uninjured hands in the modified technique group. SPSS version 17.0 (SPSS Inc., Chicago, IL, USA) was used for the statistical analyses. Differences were considered statistically significant at *P* < 0.05.

## Results

**Fig. 4:**
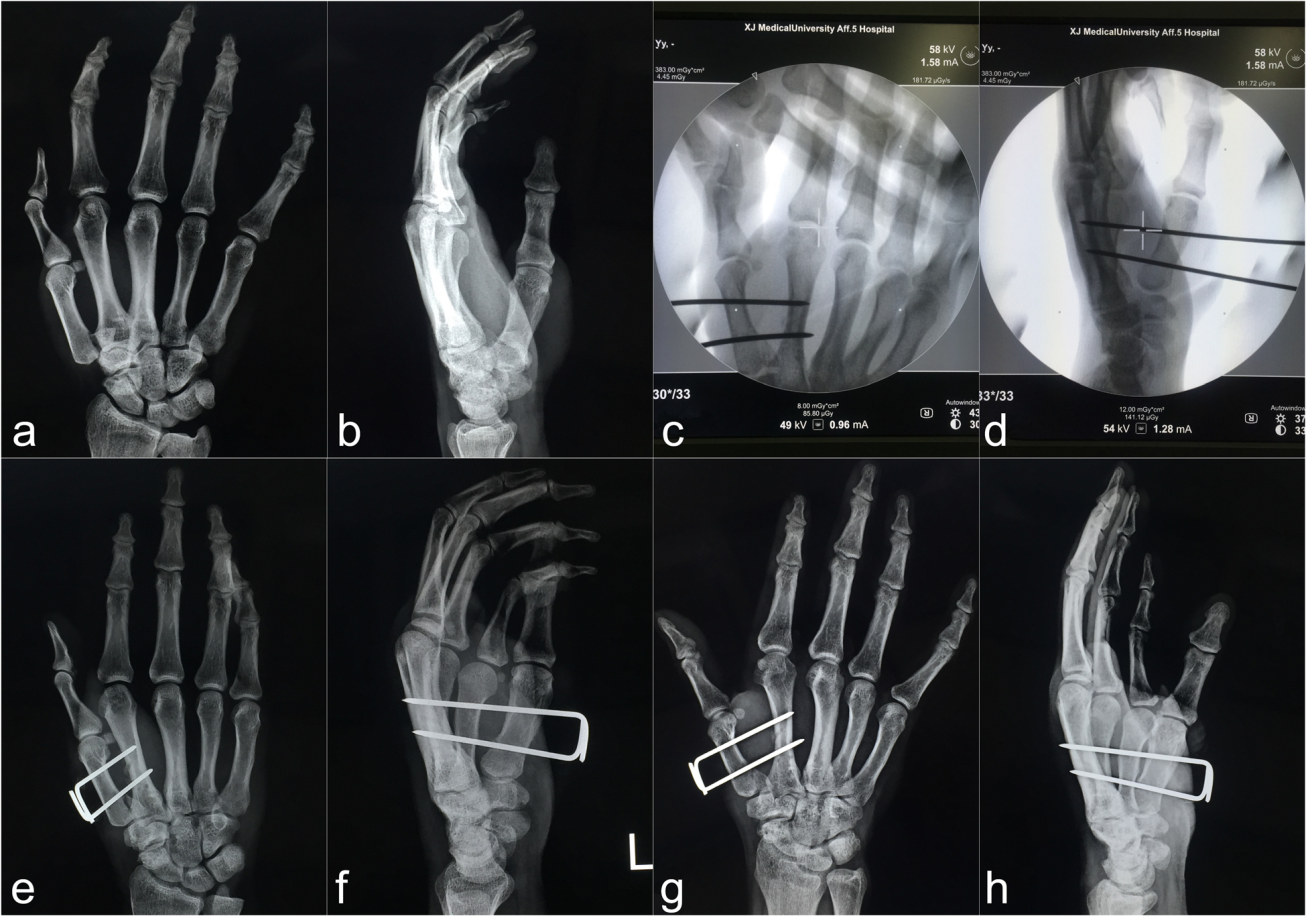
Representative case of a Bennett fracture in the modified technique group (Patient 8). **a, b** Preoperative radiographs. **c, d** Fluoroscopic images after intraoperative reduction and fixation. **e, f** One-day postoperative radiographs. A 2-mm step and a 1-mm gap were present in the articular surface. **g, h** Six-week postoperative radiographs before the K-wire was removed. The fracture had almost healed, the gap between the fracture blocks had disappeared, and the articular step was not obvious

All patients underwent satisfactory reduction and fixation by closed means. Only one Bennett fracture had a 2-mm step and a 1-mm gap in the articular surface after surgical reduction (Fig. 4).

The operation time was significantly shorter in the modified technique group than the traditional technique group (Table 3). The mean follow-up time was 13 months (range, 10–18 months). Twenty-two patients were followed up in the hospital, while eight patients were followed up by telephone and WeChat video; the patients who were not followed up in the hospital were mailed a protractor to measure the range of motion of the thumb, and a dynamometer to measure the grip and pinch strength. No infections occurred postoperatively, no patients developed K-wire loosening or fracture displacement, or malunion during the functional exercises, and there was no delayed union or nonunion (Tables 1 and 2). The plaster was removed 3 to 5 weeks after the operation, and the K-wires were removed only when the fracture had stabilised and partially healed as shown on radiographs; the removal time did not significantly differ between the modified technique group and the traditional technique group (Table 3). At final follow-up, the modified technique group had a significantly better mean VAS score and Kapandji score than the traditional technique group (Table 3). Most patients were not willing to undergo radiographic re-examination after 1 year, so the radiological evaluation results were not obtained in all cases.

**Table 3:** Outcomes of the hand function recover in modified technique and traditional technique

	Modified technique	Traditional technique	*P* value
Age (years)	35.9 ± 10.6	41.1 ± 9.1	0.196
Operation time (min)	18.3 ± 6.5	34.3 ± 8.0	< 0.001*
K-wire removal time (days)	47.6 ± 7.4	50.2 ± 5.9	0.344
Pain (VAS)	0.6 ± 0.7	1.8 ± 1.1	0.001*
Kapandji score	9.4 ± 0.7	8.1 ± 1.2	0.001*

Data are expressed as mean ± SD. *Statistically significant

VAS visual analog scale (0 ± 10)

Kapandji force (0 ± 10)

**Fig. 5:**
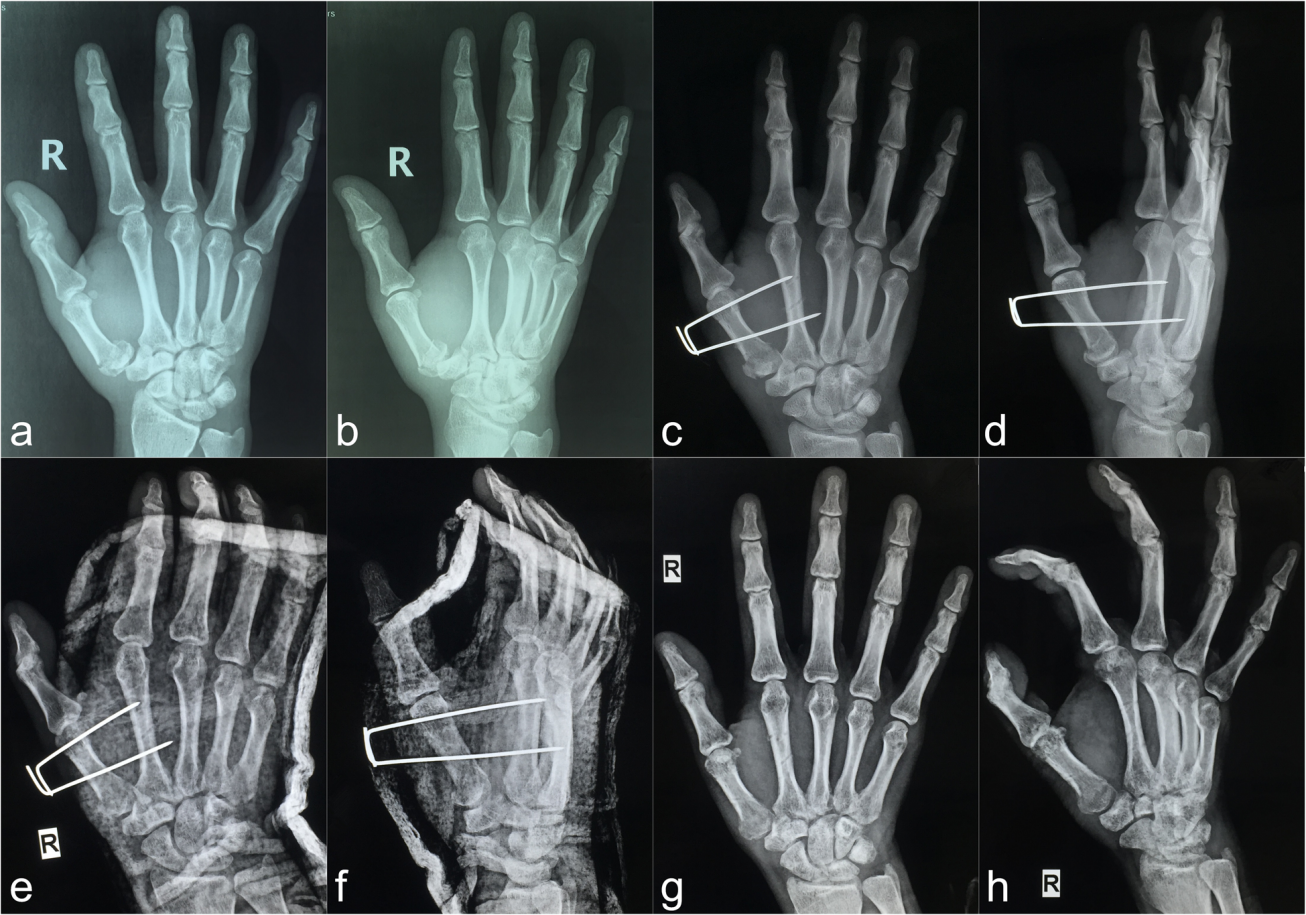
Representative case of a Rolando fracture in the modified technique group (Patient 3). **a, b** Preoperative radiographs. **c, d** One-day postoperative radiographs. **e, f** Four-week postoperative radiographs before the plaster was removed. **g, h** Eight-week postoperative radiographs after the K-wire was removed. The radiographs show that the fracture has healed

**Fig. 6:**
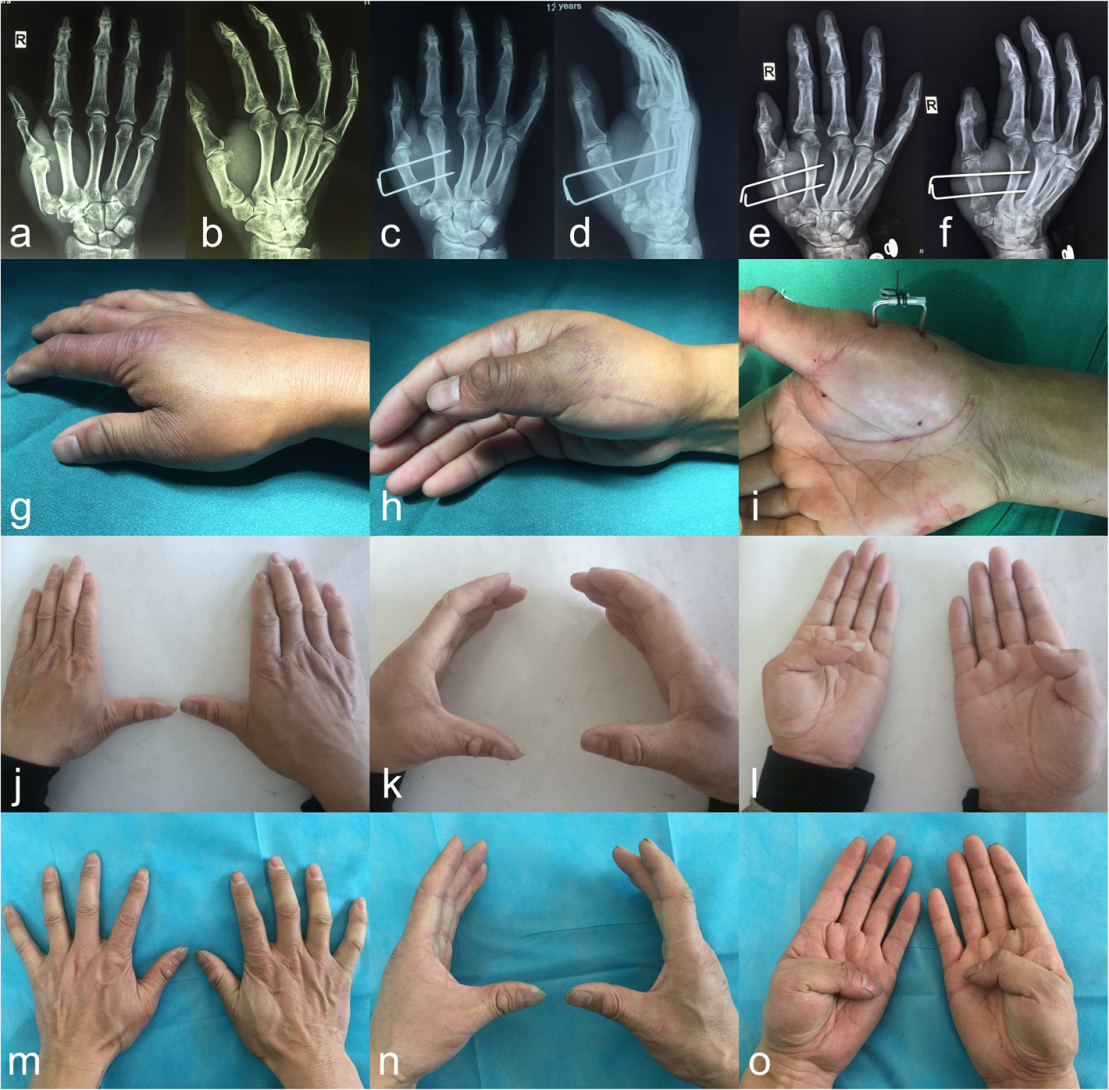
Representative case of an extra-articular fracture in the modified technique group (Patient 5). **a, b** Preoperative radiographs. **c, d** One-day postoperative radiographs. **e, f** Six-week postoperative radiographs before the K-wire was removed. **g–i** Appearance during the operation. **j–l** Functional appearance after 6 months. **m–o** Functional appearance after 1 year. The abduction and bending ability of the thumb were significantly lower on the injured than uninjured side 6 months postoperatively, but the difference was not significant 1 year postoperatively

In the modified technique group, the mean grip and pinch strength were 43.4 ± 7.0 and 9.1 ± 1.4 kg in the extra-articular fracture subgroup, and 43.0 ± 6.5 and 9.0 ± 1.1 kg in the intra-articular fracture subgroup, respectively. The mean radial abduction, palmar abduction, and flexion–extension arc were 64.1° ± 5.4°, 59.7° ± 6.1°, and 44.1° ± 4.8° in the extra-articular fracture subgroup, and 64.5° ± 3.4°, 60.4° ± 4.5°, and 43.8° ± 4.0° in the intra-articular fracture subgroup, respectively. The mean Kapandji score and VAS score were 9.5 ± 0.7 and 0.4 ± 0.5 in the extra-articular fracture subgroup, and 9.3 ± 0.7 and 0.9 ± 0.8 in the intra-articular fracture subgroup, respectively (Tables 4 and 5). The abduction and the flexion–extension arc of the thumb on the injured hand were significantly smaller than those on the uninjured hand in both the extra-articular and intra-articular fracture subgroups (*P* < 0.05), but the Kapandji score, grip strength, and pinch strength were not significantly different between the injured and uninjured hands in either the extra-articular or intra-articular fracture subgroups (*P* > 0.05) (Tables 4 and 5). All patients were able to return to their original work or hobbies; their VAS scores were all < 2, and subjective strength scores were all > 8. Representative cases are shown in Figs. 4, 5, and 6.

**Table 4:** Outcomes of the extra-articular fractures in the modified technique group

	Injured side	Uninjured side	*P* value
Radial abduction (°)	64.1 ± 5.4	68.6 ± 3.9	< 0.001*
Palmar abduction (°)	59.7 ± 6.1	64.3 ± 4.3	0.001*
Flexion–extension arc (°)	44.1 ± 4.8	49.2 ± 4.4	< 0.001*
Kapandji score	9.5± 0.7	9.7 ± 0.5	0.082
Grip strength (kg)	43.4 ± 7.0	41.7 ± 6.8	0.173
Pinch strength (kg)	9.1 ± 1.4	8.7 ± 0.8	0.126
Subjective force	9.4 ± 0.8		
Pain (VAS)	0.4 ± 0.5		

Data are expressed as mean ± SD. *Statistically significant

Strength was assumed to be 6% higher on the dominant side than the opposite side

*VAS* visual analog scale (0–10)

Subjective force (0–10)

**Table 5:** Outcomes of the intra-articular fractures in the modified technique group

	Injured side	Uninjured side	*P* value
Radial abduction (°)	64.5 ± 3.4	68.0 ± 3.9	0.003*
Palmar abduction (°)	60.4 ± 4.5	64.0 ± 3.5	0.001*
Flexion-Extension arc (°)	43.8 ± 4.0	49.1 ± 4.4	< 0.001*
Kapandji score	9.3 ± 0.7	9.7 ± 0.5	0.282
Grip strength (kg)	43.0 ± 6.5	42.1 ± 6.6	0.730
Pinch strength (kg)	9.0 ± 1.1	8.6 ± 1.1	0.311
Subjective force	9.1 ± 1.1		
Pain (VAS)	0.9 ± 0.8		

Data are expressed as mean ± SD. *Statistically significant

Strength was assumed to be 6% higher on the dominant side than the opposite side

*VAS* visual analog scale (0–10)

Subjective force (0–10)

## Discussion

Treatment of a first metacarpal basal fracture is relatively difficult, and the treatment of such fractures involving dislocation is particularly challenging [16]. However, closed reduction and percutaneous K-wire fixation can obtain a satisfactory curative effect for both the Bennett fracture and Rolando fracture [17–20].

Numerous techniques have been reported for closed reduction and percutaneous K-wire fixation of first metacarpal basal fractures [4, 8–11, 21, 22]. Although the result of parallel K-wires fixation between the first and second metacarpals reported by Van Niekerk and Ouwens in 1989 seemed unsatisfactory [10], the concept of minimally invasive and reliable fixation was worthy of further investigation. This influenced the development of modified techniques that have subsequently achieved a good curative effect [11]. Our technique is a further modification of the technique involving parallel K-wire fixation between the first and second metacarpals for the treatment of first metacarpal basal fracture. The highlights of our technique are that the ends of the K-wires were bent at 90° toward each other, and the wires were then sheathed with a section of infusion tube and strapped with silk thread. This simple operation enables the parallel K-wires to form a stable frame structure similar to an external fixator; coupled with the support of the second metacarpal, a stable rectangular frame is formed.

Previous studies have reported conflicting outcomes of closed reduction and parallel K-wire fixation for first metacarpal base fracture. In Van Niekerk and Ouwens’ report [10], only 14 of the 19 intra-articular fractures were successfully treated by closed reduction and parallel K-wire fixation, and three of the 23 patients had poor recovery that affected their daily life and hobbies. In contrast, Greeven et al. [11] reported that they achieved good results with the application of this technique; only one patient with an extra-articular fracture experienced functional limitations and could not engage in their previous hobby, but was able to return to work. The follow-up results obtained by Greeven et al. [11] were obviously better than those obtained by Van Niekerk and Ouwens [10], who performed this surgical technique earlier. This difference may be related to the maturity of the surgical techniques.

In our study, all 20 patients who underwent the modified surgery were able to return to their original work or previous activity or hobby, and although their thumb movement declined after 1 year, they were satisfied with the strength, range of motion, and symptoms of their hand. By comparison, two of the 10 patients who underwent traditional surgery developed joint stiffness that prevented them from pursuing their previous hobbies. The good clinical results in the modified technique group may be related to our modification of the surgical technique, the bending of the K-wires, and the interlocking fixation, which enhanced the stability; thus, postoperative plaster fixation could be largely avoided (the rate of plaster use in our modified technique group was 2/20 (10%), compared with 12/25 (48%) in the report by Greeven et al. [11]). Therefore, the functional exercises performed immediately postoperatively promoted rehabilitation of our patients’ hand function (Fig. 3).

In the traditional technique, Wagner’s method [4] requires the K-wire across the first carpometacarpal joint, which is also unavoidable in the cross K-wire fixation technique. However, this transarticular K-wire fixation may damage the joint surface, which occurred in almost all of the traditional surgeries included in our study. A high incidence of traumatic arthritis has been reported for transarticular K-wire fixation (16 of 21patients) [7]. Moreover, it is difficult to drive the K-wire diagonally into the first metacarpal base or the fracture blocks, and repeated drilling may damage the joint and cause a new fracture. Therefore, repeat manipulation and fluoroscopy are needed to determine the correct position during traditional surgery; thus, the operation time in the group that received the traditional technique was longer than that of the group that underwent modified technique. In addition, only performing fixation of the bases of the first and second metacarpals and/or fixation of the first carpometacarpal joint is still unstable, and so postoperative plaster-assisted fixation is necessary [5]. In contrast, our modified technique of drilling parallel K-wires through the first and second metacarpals is easy to perform; we only need to fix the proximal and distal ends of the first and second metacarpal with K-wires, which does not have strict requirements as to the position of the K-wires. By strapping the bending ends of the two K-wires, this forms a stable rectangular frame so that the length of the first metacarpal is maintained and the axial rotation is resisted; thus, it is more stable than longitudinal K-wire fixation [23]. Furthermore, our technique of parallel K-wire fixation avoids direct damage to the joint, and does not interfere with the blood supply to the fracture; thus, our technique may achieve a higher rate of fracture healing than other K-wire fixation techniques or plate fixation, although this has not yet been investigated. Greeven et al. [11] reported that three of 25 cases developed needle infection and were cured by oral antibiotics. However, none of the 30 patients in our study developed an infection. This difference may be related to the health education of the patients and the strict bandaging and care of the exposed K-wires after the surgery. Potenza et al. [24] also achieved satisfactory results when they applied this parallel K-wire fixation technique in the treatment of fifth metacarpal neck fractures.

Duan et al. [22] recently introduced a frame structure to treat comminuted fractures of the first metacarpal base. The structure was made of multiple K-wires and bone cement and also served as a type of external fixator. However, the operation was complex, it was difficult to avoid damaging the metacarpals, and it was difficult to maintain the fracture reduction while the cement set; moreover, the appearance was cumbersome, and the application of bone cement increased the medical costs (extra expenses 110 USD). Adi et al. [8] reported the use of trapezoidal K-wire fixation between the first and second metacarpals in the treatment of first metacarpal base fractures. The K-wires were also bent and fixed, but the fixation was achieved using a special locking device. However, it was difficult to avoid K-wire deviation and slippage when they were drilled into the metacarpal shaft at an acute angle, so the operation time is longer (mean tourniquet time 30 min). Additionally, the use of special locking devices increased the medical cost (nearly 100 USD), and the removal was inconvenient. Similarly, Shafific [25] used a self-made external fixator (parallel K-wires) for metacarpal and phalanx fractures and performed the locking fixation of the K-wires using a commercial device (locking ball/Jurgan ball), but the medical costs were substantial, and the fixation and subsequent removal operations were not easy. In contrast, our method is easy to perform, achieves good stability, is very cheap (owing to the use of infusion tube and silk thread), and only requires stitch cutting to remove the K-wires.

The only problem with our technique is that movement of the first metacarpal can be restricted by the fixation, and the thumb motion may therefore decline after the surgery [10]. This phenomenon was observed in our study, and the solution is to open the thumb and maximise the space between the first and second metacarpals during the operation [8].

Our study had several limitations. First, this was a retrospective, observational trial with a relatively small sample size, and the follow-up was short. Second, although we evaluated the efficacy of the modified technique by comparing it with the traditional K-wire fixation technique, a randomized control group could not be created. Due to the poor stability of the traditional technique, plaster fixation is still indispensable, so it is not the most ideal control model (with more different parameters). In addition, we mainly obtained data regarding the surgical effect in the modified technique group through comparison of the injured and uninjured hands, and although we adjusted for the dominant hand in the data analysis, we still cannot exclude the influence of the dominant hand. Third, traumatic osteoarthritis was not evaluated because 1-year postoperative radiographs were unavailable for some patients, and evaluation of this complication requires long-term follow-up to be meaningful [26]. This is worthy of discussion in future studies.

## Conclusion

Our modified percutaneous parallel K-wire fixation technique is a simple and effective method for the treatment of first metacarpal base fractures. The K-wire interlocking fixation technique resists axial rotation and maintains stability, which is beneficial for postoperative hand exercises and function recovery.

## Supplementary Information

**Additional file 1.** Anterior view of the modified surgical procedure.

**Additional file 2.** Lateral view of the modified surgical procedure.

## Data Availability

Not applicable

## Abbreviations

K-wire: Kirshner wire
VAS: Visual analog scale
